# Silybin-Functionalized PCL Electrospun Fibrous Membranes for Potential Pharmaceutical and Biomedical Applications

**DOI:** 10.3390/polym16162346

**Published:** 2024-08-19

**Authors:** Christina Spartali, Anna-Maria G. Psarra, Sotirios I. Marras, Costas Tsioptsias, Achilleas Georgantopoulos, Foteini D. Kalousi, Andreas Tsakalof, Ioannis Tsivintzelis

**Affiliations:** 1 Department of Biochemistry and Biotechnology, University of Thessaly, 41500 Larissa, Greece; 2 Department of Chemical Engineering, Aristotle University of Thessaloniki, 54124 Thessaloniki, Greece; 3 Laboratory of Biochemistry, Faculty of Medicine, University of Thessaly, 41500 Larissa, Greece

**Keywords:** silybin, electrospinning, encapsulation, drug release, cytocompatibility, antioxidant activity, antitumor activity, polycaprolactone fibers

## Abstract

Silybin is a natural flavonolignan with potential anticancer, antioxidant, and hepatoprotective properties. In the present study, various loadings of silybin (1, 3, and 5 wt%) were encapsulated in poly-ε-caprolactone (PCL) fibers by electrospinning, in order to produce new pharmaceutical composites with improved bioactive and drug delivery properties. The morphological characteristics of the composite fibrous structures were evaluated by scanning electron microscopy (SEM), and the encapsulation efficiency and the release rate of silybin were quantified using a UV-Vis spectrophotometer. The analysis of the membranes’ thermal behavior by differential scanning calorimetry (DSC) and thermogravimetric analysis (TGA) revealed the existence of interaction between PCL and silybin. An investigation of the cytocompatibility of the composite membranes revealed that normal cells displayed an unimpeded proliferation in the respective silybin concentrations; however, tumor cell growth demonstrated a dose-dependent inhibition. Furthermore, an effective antioxidant activity against hydrogen peroxide-induced oxidative stress in HEK-293 cells was observed for the prepared electrospun fibrous mats.

## 1. Introduction

**Figure 1:**
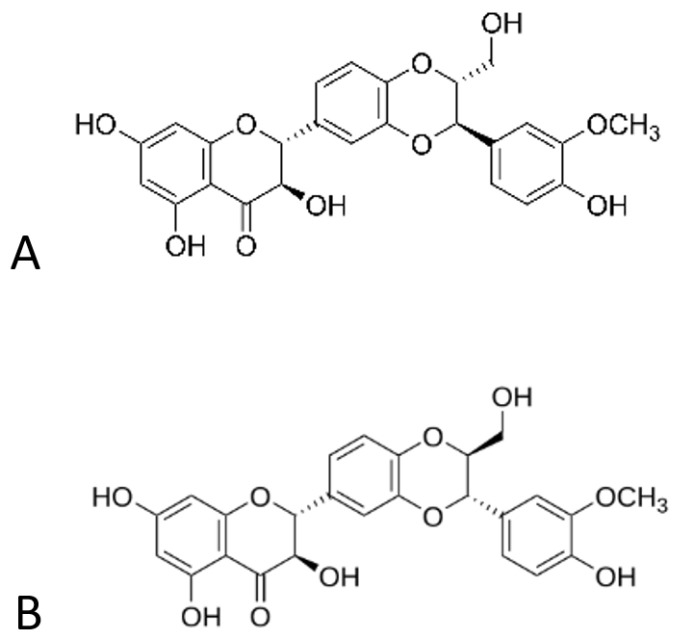
Structures of diasteisomers, silybin (**A**,**B**).

Silybin, or silibinin, is a natural flavonolignan isolated from milk thistle (*Silybum marianum*), a medicinal plant that has been used since ancient years for the treatment of numerous ailments. It has a molecular formula of C_12_H_22_O_10_, and it appears in the form of two diasteisomers, namely silybin A and silybin B (Figure 1), in a relatively quasi-molar ratio [1,2].

Silybin is the major and most biologically active ingredient of silymarin, the extract obtained from the plant’s (*Silybum marianum*) seeds [3]. It has demonstrated exceptional hepatoprotective, neuroprotective, anticancer, antioxidant, immunomodulatory, and anti-inflammatory activity, and its health benefits have been studied in comparison with other flavonoids as well [4,5,6,7]. Silybin has been used to treat a wide range of liver diseases, including chronic liver diseases, cirrhosis, and hepatocellular carcinoma, because of its anti-inflammatory, antioxidant, and antifibrotic power [8,9,10]. Moreover, it has shown a significant inhibitory effect in various types of tumors such as breast, kidney, bladder, lung, colon, and liver tumors in several in vitro and in vivo models [11,12,13]. Moreover, it exhibits neuroprotective activities against neurologic diseases, including Alzheimer’s and Parkinson’s diseases, and cerebral ischemia, possibly by inhibiting oxidative stress in the brain [14,15]. However, the low water solubility, reduced bioavailability, and short half-life of silybin significantly limit its therapeutic effect [16].

Several approaches have been investigated to develop new silybin formulations with improved pharmaceutical properties. These formulations comprise silybin containing nanoemulsions [17,18,19,20,21], liposomes [22,23,24,25], self-microemulsifying drug delivery systems (SMEDDSs) [26,27,28], and self-nanoemulsifying drug delivery systems (SNEDDSs) [29,30], encapsulation in nanostructured lipid carriers [31,32,33], and biodegradable polymer-based nanoparticles [34,35,36,37], leading to controlled and stimuli-responsive release, targeted drug delivery, enhanced bioavailability, long-term stability of the drug, the inhibition of drug toxicity and improvement in the drug thermal stability, thus broadening its application field in biomedicals, pharmaceuticals, cosmetics, and functional foods [35,38,39]. In this direction, electrospinning is a simple, cost-effective, and established technique capable of producing drug-loaded polymeric fibers with average diameters in the range of a few micrometers to submicrons or nanometers, using electrostatic forces [40,41]. The particularly thin electrospun fibers exhibit many advantages in drug delivery, such as a large surface area, enhanced loading efficiency, and tunable morphology, leading to controlled and sustained drug-releasing characteristics [42]. In addition, porous electrospun membranes can mimic both the form and functionality of the native extracellular matrix (ECM), facilitating cell attachment and proliferation [43,44]. However, analogous to other techniques, electrospinning also shows some limitations, which are related to the slow production rate and the materials that can be used [45]. Despite the exceptional advantages of this technique and the broad applicability of the produced materials, there are only a few studies on silybin encapsulated into electrospun biodegradable polymers [46,47,48,49,50], which, however, are presented without comprehensive investigation of physicochemical and pharmaceutical properties of the prepared membranes.

The selection of the appropriate polymer is very crucial for the development of a successful drug delivery system or device [51]. The physical and chemical properties of the polymer such as solubility, degradation kinetics and degradation products’ toxicity, mechanical properties, crystallinity, and hydrophobicity must be considered as they can greatly affect the preparation method [52]. Poly-ε-caprolactone (PCL) is one of the main representative biodegradable and biocompatible polymers, as it has a wide range of applications, due to its slow degradation, good solubility, low melting point, and exceptional blend compatibility [53].

Thus, the aim of this study is to provide an in-depth investigation of the physicochemical properties, as well as the antitumor and antioxidant activities of silybin-encapsulated PCL fibrous membranes, which arise from the synergistic effect of the bioactive substance and the extremely thin electrospun fibers. For this reason, various loadings of silybin were entrapped into PCL fibers by electrospinning, and the morphology of the prepared membranes was explored by scanning electron microscopy (SEM). The thermal behavior was analyzed by differential scanning calorimetry (DSC) and thermogravimetric analysis (TGA), while the encapsulation efficiency and the release rate of silybin were quantified using a UV-Vis spectrophotometer. The antitumor activity of the composite membranes was determined by evaluating HepG2 cell proliferation via an MTT (3-(4,5-dimethylthiazol-2-yl)-2,5-diphenyltetrazolium bromide) assay. The antioxidant activity of the prepared membranes due to silybin’s presence was estimated by analyzing their protective effects against H_2_O_2_-induced oxidative injury.

## 2. Materials and Methods

### 2.1. Materials

Silybin was obtained from Santa Cruz Biotechnology Inc., Santa Cruz, CA, USA (purity > 98% as reported by the provider). Poly-ε-caprolactone (PCL), dimethylformamide (DMF), dichloromethane (DCM) dimethylacetamide (DMA), phosphate-buffered saline (PBS), isopropanol, and H_2_O_2_ were purchased from Sigma-Aldrich (St. Louis, MO, USA). Dulbecco’s modified Eagle medium (DMEM), fetal bovine serum (FBS), trypsin-EDTA, penicillin–streptomycin solution 100×, and MTT (3-(4,5-dimethylthiazol-2-yl)-2,5-diphenyltetrazolium bromide) were obtained by Biowest. Dimethyl sulfoxide (DMSO) was purchased by Panreac Applichem (Barcelona, Spain). All reagents used in this work were of analytical grade and were used without further purification.

Two cell lines (HEK-293 and HepG2) were used for the biological assessment and obtained from the American-Type Culture Collection (ATCC).

### 2.2. Electrospinning

PCL solutions 8.7% *w*/*v* were prepared in a DMF:DCM (25:75 *v*/*v*) solvent system. Different concentrations of silybin were mixed with the polymer solution to prepare the silybin-encapsulated fibrous mats.

The electrospinning apparatus is a homemade setup. The system includes a variable high voltage 0–30 kV power supply (Spellman High Voltage DC Supply, model RHR30P30, New York, NY, USA), a syringe pump (Harvard Apparatus, model 2274, Holliston, MA, USA) for the injection of the polymer solution, and a rotary drum covered with aluminum foil as a grounded substrate for the collection of the fibers. All experiments were performed using a 5 mL internal volume glass syringe with a needle of 1 mm diameter. All experiments were performed at ambient temperature.

The flow rate was set at 0.6 mL/h and was controlled with a syringe pump. The applied voltage was 14 kV, and the distance between the needle tip and the collector was 7 cm. All prepared samples were kept for 1 h at 40 °C in a vacuum chamber (320 Pa, Shanghai Laboratory Instrument Work Co. Ltd., Shanghai, China) to remove the residual organic solvents and moisture.

Through this process, fibrous PCL membranes containing different amounts of silybin, i.e., 0, 1, 3, and 5 wt% of the drug (samples named “neat PCL”, “PCL/1Sil”, “PCL/3Sil”, and “PCL/5Sil”, respectively) were obtained.

### 2.3. Physicochemical Characterization of the Electrospun Membranes

#### 2.3.1. Morphological Characterization

The morphology of the fibrous structures was investigated by scanning electron microscopy (JSM-6510, Jeol, Tokyo, Japan, operated at 20 kV). All surfaces were coated with graphite to avoid charging under the electron beam. The fiber size distributions were obtained by image analysis using appropriate software (ImageJ, 1.52p). In all cases, at least two different images (areas) were analyzed for each fibrous membrane.

#### 2.3.2. Thermal Analysis

The thermal characteristics of the prepared fibrous membranes were investigated using a Shimadzu DSC-50, Shimadzu Instrument Ltd., Kyoto, Japan, differential scanning calorimeter. Temperature scans were carried out at a heating rate of 10 °C min^−1^ under a constant nitrogen flow of 20 cm^3^ min^−1^. The sample weight was kept at low levels (∼2 mg) in order to minimize any possible thermal lag during the scans.

Thermal degradation studies were conducted with a Shimadzu TGA-50 thermogravimetric analyzer. Temperature scans were carried out at a heating rate of 10 °C min^−1^ under a constant nitrogen flow of 20 cm^3^ min^−1^. The samples were heated up to 600 °C.

#### 2.3.3. Encapsulation Efficiency

The amount of silybin incorporated both on the surface and inside the fibers compared to the initially added drug was evaluated using an ultraviolet–visible (UV-Vis) spectrophotometer (UV Lambda Bio+, PerkinElmer, Hopkinton, MA, USA) at 288 nm. A pre-weighed amount of the composite fibrous membrane (2–3 mg) was dissolved in a DMF:DCM (25:75 *v*/*v*) solvent system. The amount of the released silybin was quantified by UV-Vis and corresponds to the encapsulated drug. The calculated amount of drug is stated both as encapsulation efficiency and drug loading, described by Equations (1) and (2), respectively, as follows:

(1) $$Encapsulation\;efficiency\;(\%)=(\frac{Amount\;of\;encapsulated\;silybin}{Amount\;of\;silybin\;added\;initially})\;\times 100$$

(2) $$Drug\;loading\;(\%)=\frac{Amount\;of\;elcapsulated\;silybin}{Total\;weight\;of\;fibers}\times 100$$

In each case, three independent samples were analyzed each time in triplicate, and the average values (±standard deviation) were determined.

#### 2.3.4. Drug Release Study

The in vitro release of silybin was studied by incubating the composite PCL fibrous mats (12.5 mg) in 40 mL of PBS (pH 7.4) at 37 °C ± 5 °C under gentle shaking for 95 h. Aliquots of the incubated samples were withdrawn from the dissolution medium at predetermined time points for analysis, and an equal volume of fresh PBS was added for continuous incubation. The incremental release of silybin was quantified using a UV-Vis spectrophotometer (Lambda Bio+, PerkinElmer) at 288 nm and plotted as a function of incubation time using Equation (3) as follows:

(3) $$Cumulative\;drug\;released\;(\%)=\left(\frac{Amount\;of\;silybin\;released}{Total\;amount\;of\;silybin\;entrapped}\right)\times 100$$

### 2.4. Biological Characterization of the Electrospun Membranes

#### 2.4.1. Cell Culture

HEK 293 (Human Embryonic Kidney-derived) cells were cultivated in 25 cm^2^ cell culture flasks in DMEM supplemented with 10% FBS and 1% penicillin–streptomycin 100× and 2 mM L-glutamine at 37 °C in 5% CO_2_ and 95% saturated atmospheric humidity (Galaxy 170S, New Brunswick Scientific, San Diego, CA, USA). The cells were harvested from the flask once every 2–3 d after they attained the desired confluence, using trypsin–EDTA. The cell suspension was centrifuged at 1000 rpm for 5 min, and the pelleted cells were resuspended in a growth medium. A similar culturing process was followed for HepG2 cells.

#### 2.4.2. Cell Viability Assay

Cell viability was determined with the MTT (3-(4,5-dimethylthiazol-2-yl)-2,5-diphenyltetrazolium bromide) assay. This assay is a redox indicator dye that forms purple, insoluble formazan crystals upon reduction in the presence of metabolically active cells. HEK-293 cells were seeded in flat bottom 96-well plates with a density of 10,000 cells/200 mL per well and grown for 24 h. Then, the fibrous membranes were punched into 4 mm circular discs (approximately 0.5 mg) with a hole punch set (Paffrath 0800320, Le Thieulin, France), UV sterilized for 1 h (1/2 h for each side) under a laminar flow hood (Telstarav-30/70) and immersed into the culture media for 48 h. Cells in media without the fibrous material served as positive controls and were treated under identical conditions. Subsequently, 22 μL of 5 mg/mL MTT reagent was added in each well, followed by 3 h incubation. Then, culture media were carefully removed, and 150 mL of isopropanol was added into each well to dissolve the insoluble formazan crystals. Aliquots (100 μL) of the dissolved formazan solution were transferred to a new 96-well plate, and the absorbance was measured at 570 and 690 nm (for background absorbance) using a multimode plate reader (Enspire 2300, Perkin Elmer, Waltham, MA, USA). The final value was obtained by subtracting the background absorbance from the optical density at 570 nm. Cell viability was calculated as a percentage of the positive control as follows:

(4) $$Cell\;viability\left(\%\right)=\frac{{OD}_{t}}{{OD}_{c}}\;\times 100$$

where OD_t_ is the absorbance of cells treated with the fibrous material, and OD_c_ is the absorbance of the untreated cells. At least four different samples were analyzed for each case, and the average values (±standard deviation) were determined.

#### 2.4.3. Investigation of Hydrogen Peroxide-Induced Oxidative Damage Pattern

HEK-293 cells at a density of 10^4^ cells/200 mL per well were cultivated in a 96-well plate at 37 °C, 5% CO_2_ for 24 h. Afterward, cells were treated with various concentrations (0.1–0.8 mM) of hydrogen peroxide (H_2_O_2_, 15 μL/well) for 12 h. Normal HEK-293 cells with the absence of H_2_O_2_ addition were used as a control sample.

#### 2.4.4. Protective Effects of Silybin against H_2_O_2_-Induced Oxidative Stress in HEK-293 Cells

HEK-293 cells were seeded in a 96-well plate of 10^4^ cells/200 mL per well and grown for 24 h. Then, sterilized fibrous membranes (4 mm circular discs) were added to the culture media for 36 h. Subsequently, H_2_O_2_ (at concentrations 0.3 mM and 0.5 mM) was added followed by incubation for 12 h. HEK-293 cells with the absence of fibrous membranes and H_2_O_2_ treatment were used as a control sample. Cells treated with H_2_O_2_ without the addition of electrospun membranes served as a positive control (oxidative stress-subjected sample). The effect of variable concentrations of silybin on the viability of HEK-293, post-treated under different H_2_O_2_ concentrations, was evaluated by the MTT assay as described above.

## 3. Results and Discussion

### 3.1. Physiochemical Characterization of Silybin-Encapsulated Fibrous Membranes

#### 3.1.1. Morphology of Fibrous Membranes

**Figure 2:**
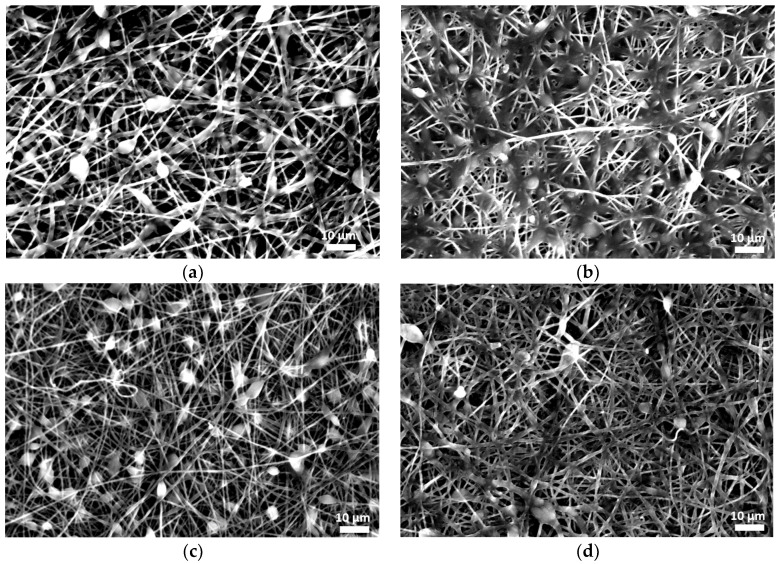
Scanning electron micrographs of the electrospun fiber morphology of (**a**) neat PCL and composite PCL fibers with (**b**) 1 wt%, (**c**) 3 wt%, and (**d**) 5 wt% silybin.

In order to study the effect of drug incorporation on the final fibrous structure, electrospun membranes were produced using neat and composite PCL with a silybin content ranging between 1 and 5 wt%. Representative images of fiber morphology are presented in Figure 2. In all cases, cross-sectionally round fibers with a minor number of beads were obtained.

**Figure 3:**
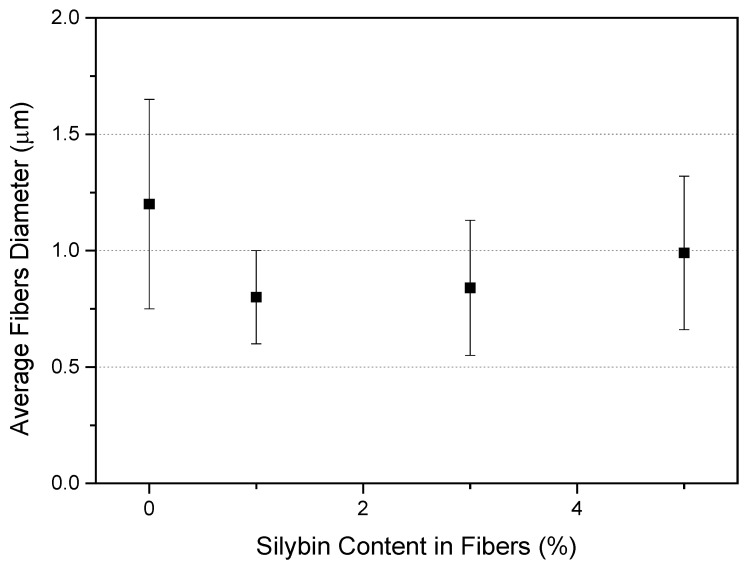
Effect of drug addition on the average diameter of electrospun PCL fibers (error bars denote the standard deviation of the fiber size distribution).

The average diameters of the neat PCL fibers were 1.20 ± 0.45 µm, while those of the silybin-entrapped PCL fibers ranged between 0.8 and 1.0 μm, as shown in Figure 3. The addition of the drug did not significantly change the morphology and the average fiber diameter.

#### 3.1.2. Thermal Behavior of Fibrous Membranes

**Figure 4:**
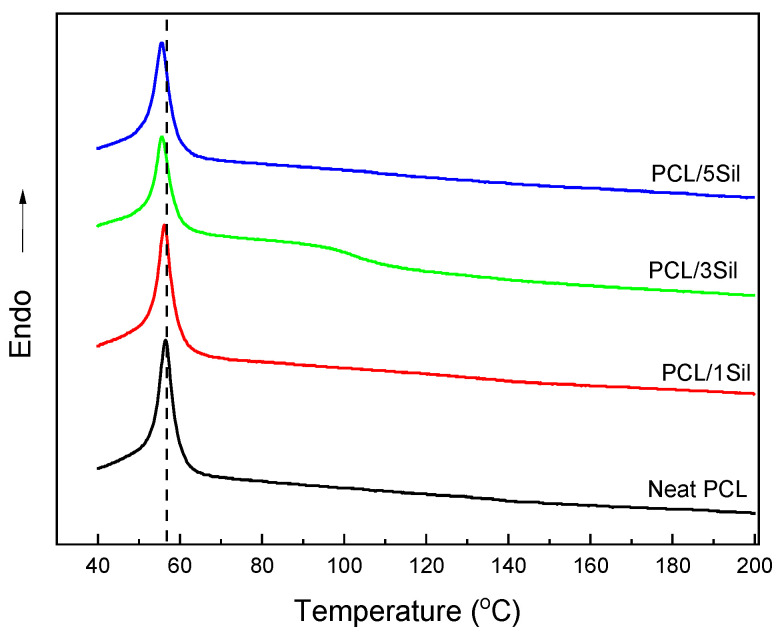
DSC heating thermograms of electrospun PCL fibers with various loadings of silybin (0, 1, 3, and 5 wt%).

Figure 4 depicts the DSC thermograms for neat and composite fibrous membranes. All samples exhibit an endothermic peak, which is attributed to the melting of the polymer. The incorporation of silybin led to a shift in this peak toward lower values. The encapsulation of small molecules into the polymer matrix can impede crystallization by acting as a physical hindrance to the diffusing polymer molecules that travel to the growing crystallite, thus promoting the melting process. Similar behavior of electrospun composite fibers has also been reported [54]. This hindrance may arise from hydrogen bonding between the hydroxyl groups of silybin and the oxygen atoms of PCL.

**Figure 5:**
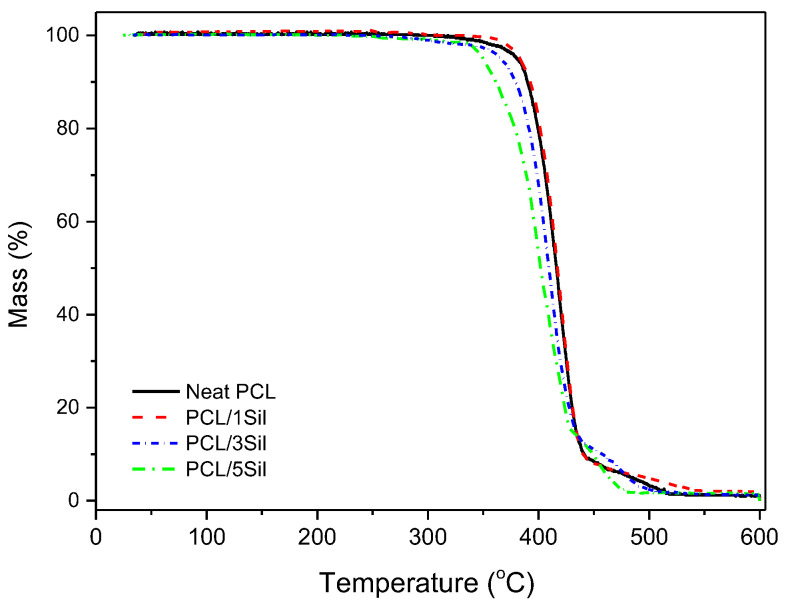
TGA curves for neat and composite electrospun PCL fibers (with 0, 1, 3, and 5 wt% silybin) obtained in an inert atmosphere.

Figure 5 shows the TGA data for the neat and composite fibrous membranes. Neat PCL practically exhibits one-stage decomposition initiated at approximately 310 °C, and the composite membranes exhibit two-stage decomposition, one at approximately 280 °C (where silybin exhibits the maximum decomposition rate) and one in the temperature range of 320–350 °C [55]. It is worth mentioning that silybin does not exhibit neat melting but melting accompanied by a partial decomposition around 150 °C [55]. Thus, the above-mentioned decrease in the temperature of decomposition initiation could be attributed to the interaction of PCL with the partially decomposed silybin. The partially decomposed silybin, most likely, also interacts with PCL via hydrogen bonding, which leads to a small weakening of chemical bonds, which in turn results in decreased thermal stability [55]. The interaction of silybin and PCL most likely affects the release profile of silybin inside water. The phase behavior of the ternary system PCL–silybin–water could be used for the design of smart nanobiocomposites [53]. More precisely, among these three compounds, there is a large number of available interactions. The equilibrium of this system is influenced by various factors such as temperature, pH, ionic strength of the aqueous phase, swelling of the polymer matrix, etc. By choosing appropriate conditions, the composition of the PCL-rich phase and the composition of the water-rich phase may be tuned, and thus a desirable release profile may be obtained. However, this is not an easy task, since most of those factors, such as the composition of the aqueous phase, the temperature, and the pH, are imposed by the relevant biomedical application and cannot be effectively changed.

#### 3.1.3. Encapsulation Efficiency and Drug Loading

Table 1 presents the entrapment efficiency and the % loading efficiency of silybin into the electrospun PCL fibers. As shown, the total percentage of drug (encapsulated inside the fibers and adsorbed on the surface of the fibers) in the samples varies between 89 and 93%.

#### 3.1.4. In Vitro Release of Silybin

**Figure 6:**
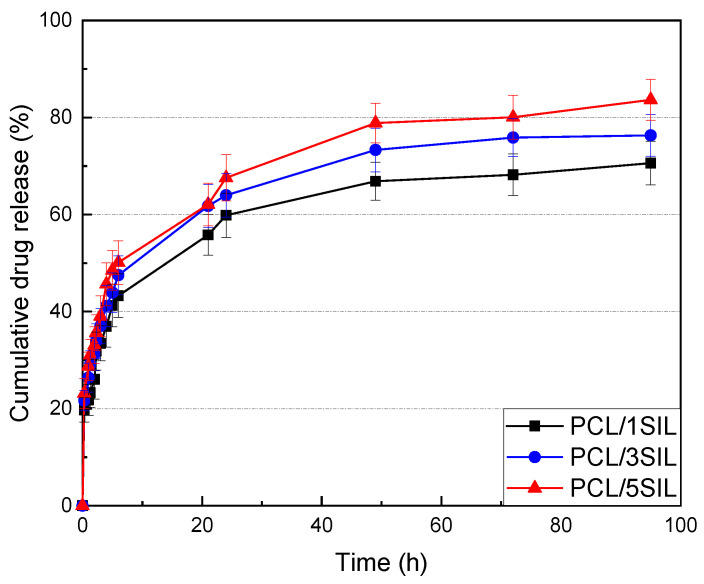
In vitro drug release profiles from silybin-encapsulated PCL fibrous membranes (with 1, 3, and 5 wt% silybin) at different time intervals.

The sustainability of silybin encapsulated into the PCL fibers was investigated by an in vitro drug release assay in a pH 7.4 phosphate buffer medium at a controlled temperature of 37 °C. The accumulative amount of silybin released from the drug-loaded PCL fibers is depicted in Figure 6. The release rate of silybin was found to be increased with enhancing drug content in electrospun fibers. Thus, the release percentages for the prepared samples with 1, 3, and 5 wt% silybin after 24 h were 59.8%, 63.9%, and 67.5%, respectively. The release profiles present an initial burst release followed by a gradual increase and reach a plateau at around 24 h (Figure 6).

The initial sharp increase could be attributed to the amount of silybin that is adsorbed on the surface of the PCL fibers or is loosely bound near the surface, which diffuses rapidly into the release medium, leading to an uncontrolled release [56]. Fast solvent evaporation during the preparation of drug-loaded fibers causes a heterogeneous distribution of the drug in the fibers. Thus, a drug concentration gradient occurs along the radius of the polymer fiber. When the composite membrane is immersed into the release medium, a higher concentration of drug entrapped at the periphery of the fiber diffuses out.

Regarding the part of the drug entrapped into the polymer matrix, it must diffuse through the polymer matrix before being released into the bulk. The rate of drug release indicates a number of processes, which include the rate of medium diffusion into the PCL matrix, drug partitioning in the polymer–buffer medium system, drug solubility, and diffusivity of the drug in the polymer [57]. For PCL, which is a biodegradable polymer, fibers’ geometry changes with time, and the average distance of diffusion will alter depending on the rate of polymer degradation. Another important parameter that can affect the release behavior is the compatibility between the bioactive substance and the polymer, as well as the drug solubility in the solvent. Strong interactions between drug molecules and polymer chains and the increased solubility of the drug in the polymer could lead to a better distribution of the drug, thus restricting the burst release phenomenon [58].

### 3.2. Biological Characterization of Silybin-Encapsulated Fibrous Membranes

#### 3.2.1. Biocompatibility Evaluation

**Figure 7:**
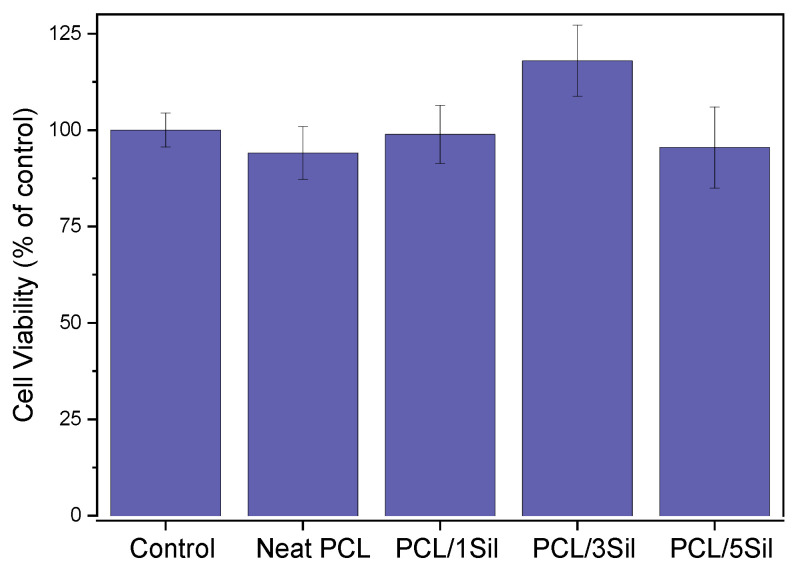
Effect of neat and silybin-loaded PCL fibers (with 0, 1, 3, and 5 wt% silybin) on HEK-293 cell viability. Cells were cultured for 48 h in the presence of fibrous membranes, and cell proliferation was evaluated by the MTT assay. Data are expressed as mean ± standard deviation (*n* = 4–5).

For use in biomedical applications, it is crucial to ensure the cytocompatibility of the prepared materials. An investigation of the composite membranes’ toxicity was conducted by evaluating HEK-293 cell viability via the MTT assay. Cells were cultured for 48 h in the presence of fiber mats, and the results of cell viability analysis are presented in Figure 7. As can be noticed, cells displayed an unimpeded proliferation in the respective silybin concentration used. Cells treated with PCL/3SIL fiber mats exhibited the highest cell viability; however, the difference, compared to the control, was not statistically significant. Nevertheless, the speculated small increase could be possibly attributed to the antioxidant and anti-inflammatory actions of silybin, which are beneficial to cell growth.

#### 3.2.2. In Vitro Antitumor Activity

Silybin has been extensively investigated for its biochemical activities, including its positive effects in both cancer prevention and treatment. Studies have confirmed that it can be an effective agent against various cancer types such as liver cancer [59], prostate cancer [60], skin cancer [61], colorectal cancer [62], breast cancer [63], gastric cancer [64], cervical and ovarian cancer [65], lung cancer [66], and bladder cancer [67]. The antitumor activity of silybin is attributed to different mechanisms such as the inhibition of cell division and proliferation factors, growth factors, cell mitogen controllers, antiapoptotic proteins, and cell cycle regulators, along with the enhancement of apoptosis inducers and growth blockers [11].

**Figure 8:**
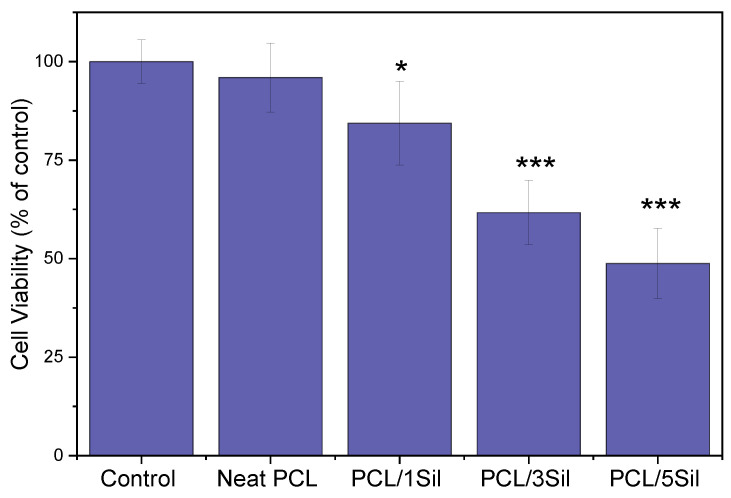
Effect of neat and silybin-loaded PCL electrospun fibers on the cell growth of the carcinoma cell line (HepG2). Cells were cultured for 48 h in the presence of fibrous membranes, and cell viability was evaluated by the MTT assay. Data are expressed as mean ± standard deviation (*n* = 4–5); * *p* < 0.05, *** *p* < 0.001 compared to controls.

Figure 8 shows the cell viability of the carcinoma cell line HepG2. Neat PCL fibers did not exhibit antitumor characteristics as they did not significantly affect cell growth and proliferation. By contrast, silybin-loaded fibers showed a statistically significant antitumor activity in a dose-dependent manner. Thus, inhibition rates of 15.6%, 38.4%, and 51.4% were observed for the hepatoblastoma cell growth treated by PCL fibrous mats with 1, 3, and 5 wt%, respectively, after 48 h cultivation. The induction of HepG2 cell apoptosis due to silybin presence has been highlighted by several researchers [68,69].

A comparison of Figure 7 and Figure 8 reveals that silybin causes a drastic reduction in tumor cell proliferation without having a toxic effect on normal cells. Similar results have been reported by other researchers who investigated milk thistle extract activity on cancer cells as well as their normal counterparts [47,70].

#### 3.2.3. Antioxidant Activity of Fibrous Membranes in H_2_O_2_-Induced Oxidative Stress

**Figure 9:**
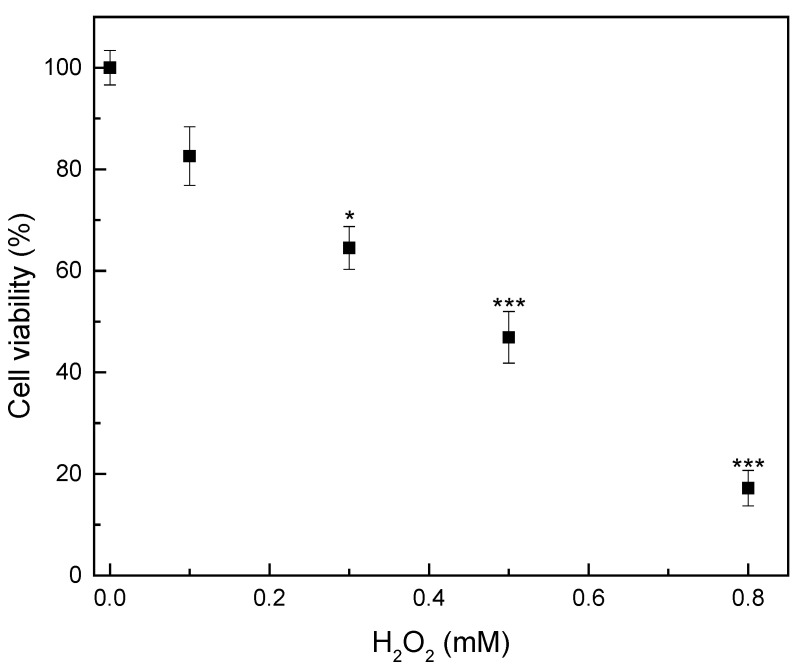
Effect of various H_2_O_2_ concentrations on the viability of human embryonic kidney-293 cells. Cells were treated with H_2_O_2_ for 12 h. Cell viability was assessed by the MTT assay. Data are expressed as mean ± standard deviation (*n* = 4–5); * *p* < 0.05, *** *p* < 0.001 compared to control (0 mM H_2_O_2_).

Oxidative stress is a phenomenon related to a disturbance in the balance between the production and accumulation of oxygen-reactive species in cells and tissues and the ability of a biological system to develop antioxidant defenses. It is considered to be one of the main contributing factors to neurodegenerative, kidney, lung, eye, heart, liver, skin, and reproductive system diseases [71]. In the present study, the capability of the composite membranes to scavenge free radicals induced by H_2_O_2_ in a cell culture model was investigated. Figure 9 presents the cytotoxicity effect of different concentrations of H_2_O_2_ (0.1–0.8 mM) on the viability of the HEK-293 cells. As can be observed, cell viability displays a dose-dependent decrease. H_2_O_2_ presence produces highly reactive hydroxyl radicals that attack essential cell components such as DNA, membrane lipids, proteins, and carbohydrates, leading to many pathological processes [72]. Thus, cell viability decreased to 82.5% for a H_2_O_2_ concentration of 0.1 mM, which is the minimum dose, while it decreased to 17% when the maximum dose was applied. A similar effect of H_2_O_2_ on the viability of human embryonic kidney HEK-293 cells was also noticed by other researchers [73,74].

**Figure 10:**
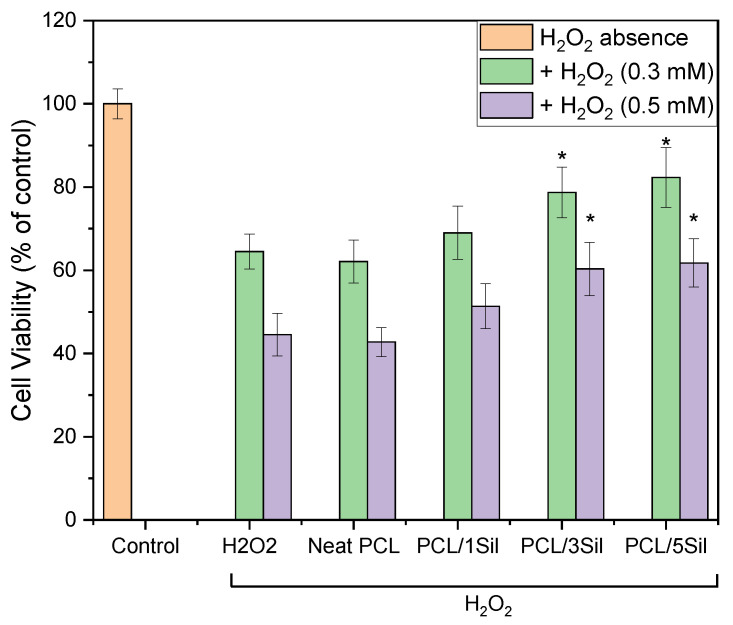
Protective effect of silybin against H_2_O_2_ oxidative damage on HEK-293 cells. Cells were treated with drug-loaded membranes for 48 h. The viability of cells after H_2_O_2_ treatment of 12 h was assessed by the MTT assay. Data are expressed as mean ± standard deviation (*n* = 3–5); * *p* < 0.05, compared to the H_2_O_2_-treated cells without protection.

Subsequently, two intermediate concentrations of H_2_O_2_, 0.3 and 0.5 mM, were selected as the optimal damage concentrations to evaluate the protective effect of silybin-loaded PCL fibrous membranes. Figure 10 shows that when HEK-293 cells were exposed to the aforementioned H_2_O_2_ concentrations without the presence of composite fibrous membranes, a substantial decrease in cell viability was observed compared to the control samples. However, when cells were pretreated with the drug-loaded membranes, an increase in cell viability in comparison with the injured group was observed. Silybin presence could lessen the H_2_O_2_-induced damage in a dose-dependent way, showing important protective effects, particularly when silybin concentration was 3 wt% or higher (*p* < 0.05).

The role of silybin in suppressing oxidative stress has been highlighted by many researchers [75,76]. Furthermore, the protective properties of silybin have been related to the antioxidant and free radical scavenging activity that it exhibits [75,77,78]. The mechanisms of silybin antioxidant defense may include the direct scavenging of free radicals [78]; having a protective effect on the structure and function of mitochondria; the hindrance of enzymes responsible for ROS production (such as xanthine oxidase and NADPH oxidase) [79]; supporting the activity of antioxidant enzymes and non-antioxidants; preserving an optimal redox equilibrium in cells [80]; the activation of the vitagene network, a group of genes that are involved in preserving cellular homeostasis during stressful conditions; enhancing protective properties [81]; and having an impact on the gut microenvironment, which is the place that the antioxidant action of polyphenols occurs [75].

## 4. Conclusions

In the present study, novel pharmaceutical formulations of silybin were prepared by encapsulating various loadings of the drug (1, 3, 5 wt%) into fibrous PCL membranes using the electrospinning technique. The use of this method ensured the high efficiency of silybin encapsulation and the production of membranes with the sustained release of the drug. The novel formulations are also biocompatible and nontoxic, as demonstrated by the MTT assay on HEK-293 cells. At the same time, they have selective antitumor and antioxidant properties, which depend on the amount of encapsulated silybin. In particular, inhibition rates of 15.6%, 38.4%, and 51.4% were recorded for the human hepatoblastoma HepG2 cell proliferation treated by PCL fibrous mats containing 1, 3, and 5 wt% of the drug, respectively, after 48 h cultivation. In addition, composite membranes can protect HEK-293 cells against oxidative damage and apoptosis induced by hydrogen peroxide, increasing the survival rate of H_2_O_2_-treated cells (0.5 mM) to 38.5%, in the case where the highest silybin loading (PCL/5SIL) was applied.

The incorporation of the drug did not considerably affect fiber morphology and their mean diameter, while thermal analysis pointed to the existence of interaction between PCL and silybin.

Further investigation of the composite membranes is currently in progress, which will provide a deeper understanding of the polymer–drug interactions and the membranes’ behavior.

## Figures and Tables

**Table 1:** Encapsulation efficiency and silybin loading for the composite fibrous membranes.

Samples	Encapsulation Efficiency ^1^ (%)	Loading ^1^ (wt%)
PCL/1Sil	93 ± 3	0.9 ± 0.1
PCL/3Sil	90 ± 2	2.7 ± 0.1
PCL/5Sil	89 ± 3	4.5 ± 0.2

^1^ Data are expressed as mean ± standard deviation (*n* = 3).

## Data Availability

Data are contained within the article.
